# How a Doctor Looks

**Published:** 1876-05

**Authors:** 


"*Three faces wears the Doctor: when first sought*
An Angel's; and a God's, the cure half-wrought;
But when, that cure complete, he seeks his fee,
Beelzebub looks less terrible than he."

